# Is the Hypodermic Injection of Piles Dangerous?

**Published:** 1879-01

**Authors:** Edmund Andrews

**Affiliations:** Prof. of Surgery in Chicago Medical College, No. 6 Sixteenth St., Chicago, Ills.


### Selections.

---

Is the Hypodermic Injection of Piles Dangerous?—About two and one-half years ago I discovered, and published to the profession, the secret method of the intinerant "pile doctors."

The plan of these itinerants, which was sold as a secret, and at a high price, from one quack to another, is substantially as follows:

A hypodermic syringe, with a very fine, sharp point, is charged with a strong solution of carbolic acid. Generally three parts of crystalized acid to one of any bland oil are employed, but they sometimes combine them in equal parts, and, for the oil occasionally substitute glycerine.

The method of the operation is to insinuate the point of the syringe into one of the piles, and throw in a few drops only of the solution. This causes the hæmorrhoid to turn of a white color and gradually to wither away. Another one is then attacked, and thus by degrees a complete cure is affected, without causing enough irritation at any one time to take the patient away from business. The operation is sometimes painless, but in other cases decidedly otherwise.

Attention was called to the seeming danger, that by this method the carbolized oil might be thrown directly into the dilated hæmorrhoidal veins. The injection of coagulants into venous enlargements of other parts of the body has, in a few cases, caused sudden death by embolism, a portion of the clots being carried to the heart and thence passing into, and blocking the pulmonary artery. It was suggested, therefore, that the injection of hæmorrhoidal veins might involve the same risk. The three groups of hæmorrhoidal veins intercommunicate, but the main outlet of the lower and middle groups is to the internal iliac and thence to the heart, while that of the upper is to the portal veins. It is conceivable that dislodged clots or globules of the injection might be swept by the current of blood to the heart, or possibly, might pass through the upper plexus into the portal vein, and be lodged in the liver.

I learn that a number of the itinerants have taken warning from my suggestions of danger, and employ a sort of clamp forceps to compress the base of the pile for a few moments at the time of the operation, thus hoping to prevent the passage of clots or globules along the veins.

This method has now been in use over three years, and has been applied to thousands of persons. If there be any actual danger in it such as is suggested by anatomy and by the danger of similar injections in other regions, the results would be manifest before now. Experience only can settle such matters. If on the other hand the method is safe, it ought at once to be adopted by the regular profession as the best method of dealing with this distressing disease.

To settle this question of danger I take the liberty, through the colums of this journal of asking every physician in the the United States, who has had opportunity to know the results of this treatment, to write me immediately, giving information on the following points.

First. How extensively has the plan been tried in your region?

Second. How far is the plan painless or otherwise?

Third. Have any sudden deaths or other alarming symptoms been known to follow its application, and if so how soon and what were the symptoms?

Fourth. Have any cases been followed by dangerous disease of the liver?

I propose to cull the information thus gathered, and communicate the results in a future article through the pages of this journal.

Please address,

EDMUND ANDREWS, M.D.,
Prof. of Surgery in Chicago Medical College,
No. 6 Sixteenth St., Chicago, Ills.

